# Physiological Mechanisms Underlying the High-Grain Yield and High-Nitrogen Use Efficiency of Elite Rice Varieties under a Low Rate of Nitrogen Application in China

**DOI:** 10.3389/fpls.2016.01024

**Published:** 2016-07-15

**Authors:** Lilian Wu, Shen Yuan, Liying Huang, Fan Sun, Guanglong Zhu, Guohui Li, Shah Fahad, Shaobing Peng, Fei Wang

**Affiliations:** National Key Laboratory of Crop Improvement, Key Laboratory of Crop Ecophysiology and Farming System in the Middle Reaches of the Yangtze River, Ministry of Agriculture, College of Plant Science and Technology, Huazhong Agricultural UniversityWuhan, China

**Keywords:** daily grain yield, Green Super Rice, grain yield, nitrogen use efficiency

## Abstract

Selecting rice varieties with a high nitrogen (N) use efficiency (NUE) is the best approach to reduce N fertilizer application in rice production and is one of the objectives of the Green Super Rice (GSR) Project in China. However, the performance of elite candidate GSR varieties under low N supply remains unclear. In the present study, differences in the grain yield and NUE of 13 and 14 candidate varieties with two controls were determined at a N rate of 100 kg ha^−1^ in field experiments in 2014 and 2015, respectively. The grain yield for all of the rice varieties ranged from 8.67 to 11.09 t ha^−1^, except for a japonica rice variety YG29, which had a grain yield of 6.42 t ha^−1^. HY549 and YY4949 produced the highest grain yield, reflecting a higher biomass production and harvest index in 2014 and 2015, respectively. Total N uptake at maturity (TN_PM_) ranged from 144 to 210 kg ha^−1^, while the nitrogen use efficiency for grain production (NUEg) ranged from 35.2 to 62.0 kg kg^−1^. Both TN_PM_ and NUEg showed a significant quadratic correlation with grain yield, indicating that it is possible to obtain high grain yield and NUEg with the reduction of TN_PM_. The correlation between N-related parameters and yield-related traits suggests that promoting pre-heading growth could increase TN_PM_, while high biomass accumulation during the grain filling period and large panicles are important for a higher NUEg. In addition, there were significant and negative correlations between the NUEg and N concentrations in leaf, stem, and grain tissues at maturity. Further improvements in NUEg require a reduction in the stem N concentration but not the leaf N concentration. The daily grain yield was the only parameter that significantly and positively correlated with both TN_PM_and NUEg. This study determined variations in the grain yield and NUE of elite candidate GSR rice varieties and provided plant traits that could be used as selection criteria in breeding N-efficient rice varieties.

## Introduction

Rice is one of the staple food crops for approximately half of the global population (Godfray et al., 2010), and rice production must increase by 70% by 2050 to satisfy the requirements of the growing world population (Koning et al., 2008; Godfray et al., 2010). Moreover, increased rice production needs to be achieved under the pressures of decreased arable land area, global climate change (Peng et al., 2004), intensified natural disasters (Tao et al., 2013), and the frequent occurrence of diseases and pests (Sheng et al., 2003). Therefore, it is imperative to develop new varieties that have a higher yield potential and improved adaptation to the environment.

Yield potential is defined as the yield of a cultivar grown in environments to which it is adapted, with nutrients and non-limiting water, as well as pests, diseases, weeds, lodging, and other stresses effectively controlled (Evans and Fischer, 1999). Cassman (1999) provided a more functional definition of yield potential, suggesting that this parameter is the yield obtained when an adapted cultivar is grown with the minimal possible stress, which is achieved by using the best management practices. In rice, yield potential has been significantly augmented, reflecting the utilization of semi-dwarf genes, heterosis, and the combination of intersubspecific heterosis and new plant types (Peng et al., 2008). In 2014, the elite super hybrid rice Y-Liang-You900 (YLY900) showed a record high yield of 15.4 t ha^−1^ (Li et al., 2014). However, the main dilemma is that new varieties that have a high yield potential were achieved using surplus nutrient application, suggesting that farmers should apply a higher amount of fertilizers than the minimum required to produce the highest grain yield in rice production (Peng et al., 2002). The performance of these newly developed high-yielding varieties under low nutrient input remains unclear.

Nitrogen (N) is a vital element for plant development and growth, and the application of N fertilizer could significantly increase yield formation (Andrews et al., 2013). From 1960 to 2012, the global N fertilizer consumption increased by 800% and the annual N consumption in China increased from 8 to 35% of the world's N consumption (data from IFA). In China, the average rate of N application in rice production is ~180 kg ha^−1^, which is 75% higher than the world average rate (Peng et al., 2002, 2006). High N fertilizer input leads to low nitrogen use efficiency (NUE) due to the rapid N losses from ammonia volatilization, denitrification, surface runoff, and leaching in the soil-floodwater system (Vlek and Byrnes, 1986; De Datta and Buresh, 1989). A low NUE results in significant environmental pollution, such as soil acidification (Guo et al., 2010), air pollution (Smil, 1999), and water eutrophication (Diaz and Rosenberg, 2008). To increase the NUE in rice production, scientists have developed a range of optimized crop management practices, such as Site-Specific N Management (SSNM, Dobermann et al., 2002), Real-Time N Management (RTNM, Peng et al., 2006), the San-Ding Cultivation Method (SDCM, Zou et al., 2006), and “Three Controls” Nutrient Management Technology (TCNM, Zhong et al., 2007).

One potential approach to reduce N fertilizer application in rice production is the development of varieties with an improved NUE (Sun et al., 2014; Hu et al., 2015). Variations in the NUE of different rice genotypes have been determined, and NUE-related traits have been evaluated for their accuracy in reflecting genotypic variation in rice NUE from 1987 to 2003 (Broadbent et al., 1987; De Datta and Broadbent, 1988, 1993; Tirol-Padre et al., 1996; Singh et al., 1998; Inthapanya et al., 2000; Ntanos and Koutroubas, 2002; Koutroubas and Ntanos, 2003). These studies reported significant differences in N uptake capacity and N use efficiency for grain production (NUEg), suggesting candidate parameters reflecting NUE variation, such as WP/Nt (panicle weight/total N uptake), NPI (the product of grain yield at zero N treatment and NUEg), among others. Since 2003, there have only been a few studies on NUE variation in rice. Recently, Ju et al. (2015) compared the grain yield of two N-efficient varieties and two N-inefficient varieties under low N input conditions, reporting that a high grain yield at a low N rate was associated with deeper roots, increased root oxidation activity, and a higher photosynthetic NUE. However, NUE differences among newly developed elite varieties under low N input condition have not been studied.

Zhang (2007) proposed strategies for developing Green Super Rice (GSR) to meet the challenges in rice production. In 2010, the Ministry of Science and Technology of China launched a mega project to develop GSR as proposed by Zhang (2007). One main aspect in this project is to decrease N fertilizer application in rice production through the genetic development of N-efficient varieties. Thus, the objectives of the present study were to evaluate the grain yield and NUE of the newly developed candidate GSR varieties from different breeding institutes under low N supply and to examine the physiological mechanisms underlying the differences in NUE.

## Materials and methods

### Plant materials

In 2014, 13 candidate GSR varieties were grown in the middle season with YLY6 (a super hybrid variety) and HHZ (a potential GSR) as control varieties. In 2015, 14 new candidate GSR varieties were grown in the middle season with YLY6 and HHZ as control varieties. HY549 and HLY630 were used in both years. All of the candidate GSR varieties were developed in recent years, achieving a high grain yield in local variety tests, while the two control varieties, YLY6 and HHZ, were widely planted in South China in the last decade. Detailed information concerning these varieties is shown in Table 1.

**Table 1:** **Grain yield and yield components of the varieties in 2014 and 2015 at Wuxue County, Hubei Province, China**.

**Variety**	**Abbreviation**	**Year of release**	**Type**	**Institute of release**
**2014**				
Hanyou549	HY549	–	Hybrid	Shanghai Agrobiological Gene Center
Huiliangyou858	HYL858	–	Hybrid	Anhui Academy of Agricultural Sciences
Jinkeyou651	JKY651	2013	Hybrid	Huazhong Agricultural University
9you6hao	9Y6H	–	Hybrid	Chinese Academy of Agricultural Sciences
Huiliangyou630	HLY630	2014	Hybrid	Anhui Academy of Agricultural Sciences
Rongfengyou41	RFY41	–	Hybrid	Huazhong Agricultural University
Rongyou225	RY225	2009	Hybrid	Jiangxi Academy of Agricultural Sciences
Wuyouhang1573	WYH1573	2014	Hybrid	Jiangxi Academy of Agricultural Sciences
Quanyou982	QY982	–	Hybrid	Chinese Academy of Agricultural Sciences
Yangliangyou6	YLY6	2003	Superhybrid	Lixiahe Institute of Agricultural Sciences
Huanghuazhan	HHZ	2005	Inbred	Guangdong Academy of Agricultural Sciences
Guangliangyou5	GLY5	2013	Hybrid	Hubei Academy of Agricultural Sciences
Hanyou73	HY73	2014	Hybrid	Shanghai Agrobiological Gene Center
Zhongzu14	ZZ14	2006	Inbred	Chinese Rice Research Institute
Yungeng29	YG29	2011	Inbred	Yunnan Academy of Agricultural Sciences
**2015**				
Yongyou4949	YY4949	2015	Hybrid	Ningbo Seed Company
Hanyou549	HY549	–	Hybrid	Shanghai Agrobiological Gene Center
Chuanyou5727	CY5727	–	Hybrid	Sichuan Academy of Agricultural Sciences
Jiyou225	JY225	2014	Hybrid	Jiangxi Academy of Agricultural Sciences
Yyou278	YY278	–	Hybrid	Hunan Hybrid Rice Research Center
Longliangyouhuazhan	LLYHZ	2015	Hybrid	Longping High-Tech
Huiliangyou630	HLY630	2014	Hybrid	Anhui Academy of Agricultural Sciences
Mingliangyou143	MLY143	–	Hybrid	Hunan Hybrid Rice Research Center
Luoyou10	LY10	–	Hybrid	Wuhan University
Huanghuazhan	HHZ	2005	Inbred	Guangdong Academy of Agricultural Sciences
Jinyou959	JY959	–	hybrid	Yunnan Jinrui Seed Industry Company
Zhonghua1	ZH1	–	hybrid	Guangdong Academy of Agricultural Sciences
Heliangyou7185	HLY7185	–	hybrid	Fujian Academy of Agricultural Sciences
Huhan1709	HH17-09	–	hybrid	Shanghai Agrobiological Gene Center
Shanyou108	SY108	2013	hybrid	Guizhou Academy of Agricultural Sciences
Yangliangyou6	YLY6	2003	Superhybrid	Lixiahe Institute of Agricultural Sciences
Hualiangyou1511	HLY1511	–	Hybrid	Huazhong Agricultural University
Wushansimiao	WSSM	2009	Hybrid	Guangdong Academy of Agricultural Sciences

### Experimental design

**Figure 1:**
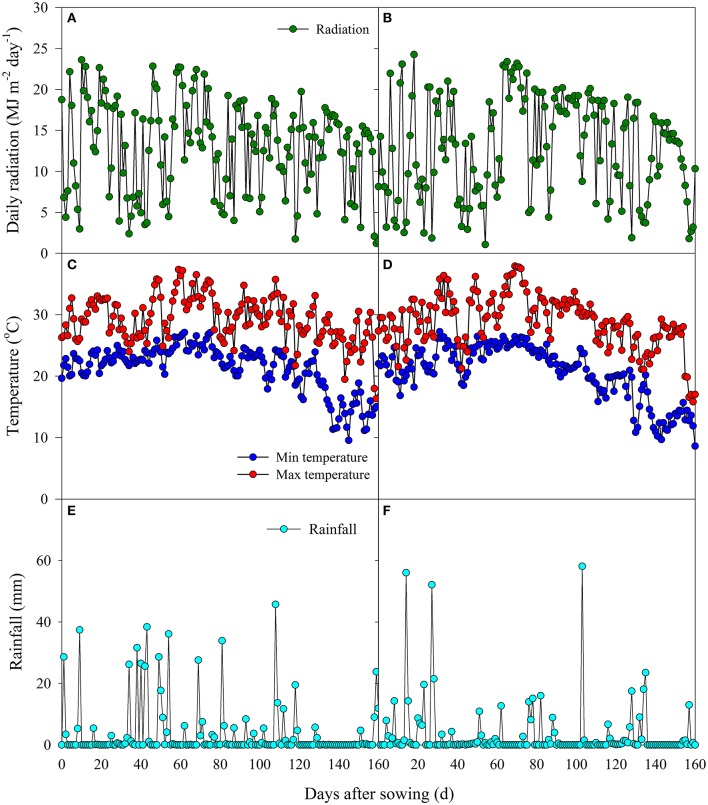
**Daily radiation, daily minimum and maximum temperatures, and daily rainfall in the middle growing season of 2014 (A, C, E) and 2015 (B, D, F)**.

Field experiments were conducted in Zhougan Village (2014) and Zhangbang Village (2015) of Wuxue County, Hubei Province, China. Prior to the experiments, soil samples from the upper 20-cm layer were collected to analyze the soil chemical properties. In 2014, the soil had a clay loam texture with a pH of 5.60, organic matter of 27.18 g kg^−1^, total N of 1.83 g kg^−1^, available P of 4.91 mg kg^−1^, and available K of 105.8 mg kg^−1^, while in 2015, the soil had a clay loam texture with a pH of 5.20, organic matter of 26.69 g kg^−1^, total N of 1.19 g kg^−1^, available P of 22.56 mg kg^−1^, and available K of 159.2 mg kg^−1^. The data for daily rainfall, solar radiation, and minimum and maximum temperatures during the rice growing season were collected at a meteorological station (CR800, Campbell Scientific Inc., Logan, Utah, USA) near the fields, and are shown in Figure 1.

The experiment was arranged in a completely randomized block design with four replications. The seedlings were raised in the seedbed with a sowing date of May 23, 2014 and May 25, 2015. Twenty-five-day-old seedlings were transplanted on June 17 and June 19 in 2014 and 2015, respectively, at a hill spacing of 20.0 × 20.0 cm with two seedlings per hill and a plot size of 30 m^2^ in 2014 and 25 m^2^ in 2015. The fertilizers were manually broadcasted and incorporated 1 day before transplanting for basal application (40 kg N ha^−1^ urea, 40 kg P ha^−1^ calcium superphosphate, 50 kg K ha^−^ potassium chloride, and 5 kg Zn ha^−1^ zinc sulfate heptahydrate for 2 years). Nitrogen topdressings were applied at midtillering (20 kg ha^−1^) and panicle initiation (PI; 40 kg ha^−1^), and K was topdressed at PI at a rate of 50 kg ha^−1^ during a 2-year experimental period. To minimize seepage between the plots, all of the bunds were covered with plastic film and installed at a depth of 20 cm below the soil surface. A water depth of 5–10 cm was maintained until 7 days prior to maturity when the fields were drained. The weeds were controlled manually and using herbicides. Pests and diseases were controlled using insecticides and fungicides; no obvious water, weed, pest, or disease stresses were observed during the experiment.

### Crop measurements

Twelve hills were sampled from each plot at mid-tillering (MT), PI, and heading (HD). Plant height and stem (main stems plus tillers) numbers were recorded. A tiller with at least one leaf was counted as a stem. The plant samples were separated into leaf blade (leaf), culm plus sheath (stem), and panicle. The green leaf area was measured using a leaf area meter (LI-3100, LI-COR, Lincoln, NE, USA) and was expressed as the leaf area index (LAI). The specific leaf weight (SLW) was defined as the ratio of the leaf dry weight to leaf area. The dry weight of each component was determined after oven drying at 80°C to a constant weight. The plant dry weight was the sum of all of the aboveground components.

At physiology maturity (PM), 12 hills were obtained from each subplot to determine the aboveground total biomass and other yield components. Plant height and panicle number were obtained from 12 hills. The plant samples were separated into leaf, stem and panicle. The dry weight of straw was determined after oven drying at 80°C to a constant weight. The panicles were hand-threshed, and the filled spikelets were separated from unfilled spikelets after submerging them in tap water. The empty spikelets were separated from the half-filled spikelets through winnowing. Three 30-g subsamples of filled spikelets, three 2-g subsamples of empty spikelets, and the total number of half-filled spikelets were obtained to quantify the number of spikelets per m^2^. The dry weights of rachis, filled, half-filled, and unfilled spikelets were determined after oven drying at 80°C to constant weight. The aboveground total biomass was calculated as the total dry matter of straw, rachis, and filled, half-filled, and unfilled spikelets. The spikelets per panicle, grain filling percentage (100 × filled spikelet number/total spikelet number), and harvest index (HI) (100 × filled spikelet weight/aboveground total biomass) were calculated. The grain yield was determined from a 5-m^2^ area in each subplot and was adjusted to a standard moisture content of 0.14 g H_2_O g^−1^ fresh weight. The grain moisture content was measured with a digital moisture tester (DMC-700, Seedburo, Chicago, IL, USA).

The tissue N concentration of each component at HD and PM was determined using an Elemental analyzer (Elementar vario MAX CNS/CN, Elementar Trading Co., Ltd, Germany). The plant N accumulation at HD and PM was calculated as the sum of N in each of the aboveground components. The nitrogen use efficiency for grain production (NUEg) was calculated as the grain yield per unit plant N accumulation. The nitrogen use efficiency in biomass production (NUEb) was determined as the ratio of biomass production to plant N accumulation. The nitrogen harvest index (NHI) was calculated as the percentage of accumulated N in grain to plant N accumulation (Peng et al., 1996).

### Statistical analysis

The data were analyzed using analysis of variance, and the mean values among the varieties were compared based on the least significant difference (LSD) test at the 0.05 probability level.

## Results

### Growth duration

The growth duration ranged from 118 to 141 d in 2014 and from 119 to 144 d in 2015. For the majority of varieties, the growth duration ranged from 130 to 141 d in 2014 and from 137 to 144 d in 2015 (Table 2). The days from sowing to flowering for these varieties ranged from 78 to 99 d in 2014 and from 84 to 101 d in 2015, and the grain filling period was from 33 to 47 d in 2014 and from 33 to 53 d in 2015. Generally, the growth duration of HY549 and HLY630 was similar in 2014 and 2015. The growth duration of HHZ in 2014 was longer than that in 2015, while the growth duration of YLY6 in 2014 was shorter than that in 2015. Notably, YY4949 had the shortest growth period prior to flowering but had the longest growth period after flowering in 2015 (Table 2).

**Table 2:** **Growth duration of the varieties in 2014 and 2015 at Wuxue County, Hubei Province, China**.

**Variety**	**Sowing-flowering**	**Flowering-maturity**	**Total growth duration**
**2014**			
HY549	99	41	141
HYL858	85	45	130
JKY651	99	38	137
9Y6H	90	47	137
HLY630	90	47	137
RFY41	85	52	137
RY225	83	54	137
WYH1573	83	47	130
QY982	85	45	130
YLY6	93	44	137
HHZ	85	45	130
GLY5	90	47	137
HY73	78	40	118
ZZ14	85	45	130
YG29	85	33	118
**2015**			
YY4949	84	53	137
HY549	95	49	144
CY5727	91	53	144
JY225	85	52	137
YY278	101	43	144
LLYHZ	95	49	144
HLY630	91	46	137
MLY143	98	39	137
LY10	92	45	137
HHZ	86	33	119
JY959	95	49	144
ZH1	95	49	144
HLY7185	87	50	137
HH1709	87	50	137
SY108	90	47	137
YLY6	98	46	144
HLY1511	95	42	137
WSSM	90	47	137

### Grain yield and yield components

The grain yield ranged from 6.42 to 10.41 t ha^−1^ in 2014 (Table 3). HY549 produced the highest grain yield, and YG29 produced the lowest grain yield. No significant difference in grain yield was observed between YLY6 and HHZ. Compared with the two controls, HY549, HYL858, JKY651, 9Y6H, HLY630, and RFY41 produced a significantly superior grain yield. The grain yields of RY225, WYH1573, QY982, and GLY5 were similar to those of the two controls. HY73, ZZ14, and YG29 produced a significantly lower grain yield compared with the two controls (Table 3). In 2015, the grain yield ranged from 8.96 to 11.09 t ha^−1^. Notably, a higher grain yield was observed in YY4949, HY549, and CY5727 than in either HHZ or YLY6. WSSM generated a significantly lower grain yield than HHZ. The average grain yield of HHZ, YLY6, HY549, and HLY630 was 9.82 t ha^−1^ in 2014 and 10.13 t ha^−1^ in 2015 (Table 3), and analysis of variance indicated that the difference in the average grain yield of the four common varieties between 2014 and 2015 was not statistically significant.

**Table 3:** **Grain yield and yield components of the varieties in 2014 and 2015 at Wuxue County, Hubei Province, China**.

**Variety**	**Grain yield (t ha^−1^)**	**Panicles m^−2^**	**Spikelets per panicle**	**Spikelets m^−2^ (× 10^3^)**	**Grain filling percentage (%)**	**Grain weight (mg)**	**Biomass (t ha^−1^)**	**Harvest index (%)**
**2014**								
HY549	10.41	180	290	52.2	83.2	23.5	18.6	54.8
HYL858	10.07	209	198	41.4	84.1	27.3	17.0	55.6
JKY651	10.05	232	176	40.9	88.9	26.0	17.4	54.4
9Y6H	10.04	183	202	37.1	90.5	28.8	17.7	54.7
HLY630	9.98	201	194	39.1	88.0	25.9	16.0	55.5
RFY41	9.98	197	194	38.1	83.2	28.3	15.9	56.5
RY225	9.71	204	231	47.1	80.0	23.1	14.9	58.4
WYH1573	9.71	208	242	50.4	89.0	20.8	16.4	57.0
QY982	9.60	207	188	38.9	87.2	29.0	17.3	56.9
YLY6	9.50	179	191	34.2	91.1	29.5	16.7	55.0
HHZ	9.39	220	219	48.1	90.6	19.5	15.0	56.5
GLY5	9.11	181	206	37.3	79.5	27.5	15.5	52.7
HY73	8.76	188	185	34.8	87.8	28.8	16.3	53.7
ZZ14	8.67	262	216	56.9	76.3	18.4	14.5	54.8
YG29	6.42	199	175	35.1	62.3	23.7	13.4	38.3
LSD (0.05)	0.39	16	17	4.0	4.2	0.5	1.3	2.0
**2015**								
YY4949	11.09	209	265	55.7	93.7	21.5	18.7	59.7
HY549	10.99	240	206	49.5	88.2	23.7	20.3	51.1
CY5727	10.93	244	168	40.9	93.2	27.8	20.3	51.5
JY225	10.65	255	173	44.1	85.2	24.2	16.4	55.5
YY278	10.60	246	180	44.2	92.6	25.3	20.3	50.9
LLYHZ	10.53	244	178	43.4	93.4	23.4	19.2	49.5
HLY630	10.49	241	172	41.6	95.8	25.9	18.1	56.9
MLY143	10.34	265	153	40.7	93.4	26.5	18.6	54.0
LY10	10.26	255	195	49.4	95.5	22.3	18.1	53.6
HHZ	10.04	277	153	42.3	93.1	21.3	16.7	50.3
JY959	9.97	202	222	44.7	90.9	24.9	19.4	51.0
ZH1	9.83	174	230	39.9	92.8	25.8	20.9	45.8
HLY7185	9.74	270	142	38.1	92.2	24.9	16.8	52.3
HH17-09	9.66	293	161	47.0	92.1	19.0	16.0	51.5
SY108	9.64	231	160	36.7	88.8	29.1	18.0	52.8
YLY6	9.61	208	170	35.3	90.4	28.6	18.4	49.5
HLY1511	9.44	214	153	32.7	92.6	28.7	17.1	51.0
WSSM	8.96	283	158	44.6	91.2	19.9	17.2	47.1
LSD (0.05)	0.92	23	36	8.2	2.5	4.0	1.9	2.1

In 2014, the higher grain yields of HY549, HYL858, JKY651, and 9Y6H primarily reflected the higher biomass production (Table 3). HY549 had a smaller number of large panicles, resulting in a significantly larger sink size (spikelets m^−2^) compared with the other varieties. In 2015, the higher grain yield of YY4949 resulted from a higher harvest index, while the higher biomass production of HY549 and CY5727 contributed to the yield advantage of these two varieties. YY4949 and HY549 had a larger sink size than the other varieties. The higher grain yields of HHZ, YLY6, HY549, and HLY630 in 2015 reflected the higher biomass production (Table 3).

### Nitrogen uptake and use efficiency

Significant differences were observed among the varieties for total N uptake at the heading stage (TN_HD_), total N uptake during the grain filling period (TN_GF_), total N uptake at maturity (TN_PM_), nitrogen use efficiency for grain production (NUEg), nitrogen use efficiency for grain production (NUEb), and NHI in 2014 and 2015. The TN_PM_ ranged from 144 to 172 kg ha^−1^ in 2014 and from 158 to 210 kg ha^−1^ in 2015. The NUEg of the varieties ranged from 35.2 to 62.0 kg kg^−1^ in 2014 and from 43.1 to 58.4 kg kg^−1^ in 2015. The TN_PM_ of HHZ, YLY6, HY549, and HLY630 in 2015 was higher than that in 2014, resulting in a lower NUEg for these varieties in 2015 than that in 2014 (Table 4).

**Table 4:** **Nitrogen uptake at the heading stage (TN_HD_), nitrogen uptake during grain filling period (TN_GF_), nitrogen uptake at maturity (TN_PM_), nitrogen use efficiency for grain production (NUEg), nitrogen use efficiency for biomass production (NUEb), and nitrogen harvest index (NHI) of the varieties in 2014 and 2015 at Wuxue County, Hubei Province, China**.

**Variety**	**TN_HD_ (kg ha^−1^)**	**TN_GF_ (kg ha^−1^)**	**TN_PM_ (kg ha^−1^)**	**NUEg (kg kg^−1^)**	**NUEb (kg kg^−1^)**	**NHI (%)**
**2014**						
HY549	173	−4.3	169	60.9	111	70.9
HLY858	137	35.4	172	55.6	101	71.6
JKY651	166	−0.7	166	57.2	105	71.6
9Y6H	156	9.0	165	59.0	108	72.9
HLY630	146	10.3	157	57.3	103	75.0
RFY41	130	32.3	162	55.6	98	71.5
RY225	113	36.6	150	58.4	100	74.6
WYH1573	132	29.1	161	58.2	102	69.5
QY982	131	28.8	160	62.0	109	75.8
YLY6	128	24.7	153	60.8	110	75.4
HHZ	121	28.7	149	57.1	100	71.3
GLY5	125	19.8	144	56.8	108	72.8
HY73	111	47.0	158	55.6	103	70.1
ZZ14	125	29.5	154	52.2	95	70.7
YG29	102	44.5	146	35.2	92	50.9
LSD (0.05)	29	36.5	18.4	5.0	7.6	4.5
**2015**						
YY4949	164	36.0	200	56.4	94	70.8
HY549	167	24.9	192	54.1	106	62.2
CY5727	177	32.8	210	49.9	97	62.8
JY225	164	19.5	184	49.5	89	63.5
YY278	161	34.4	195	53.1	104	63.8
LLYHZ	159	23.5	182	52.2	105	63.5
HLY630	155	21.9	177	58.4	103	70.5
MLY143	167	20.4	187	54.0	100	66.7
LY10	156	37.1	193	50.3	94	64.3
HHZ	165	12.0	177	47.3	94	62.5
JY959	175	17.7	193	51.6	101	65.9
ZH1	187	22.3	209	45.8	100	57.8
HLY7185	150	27.1	177	49.4	94	62.8
HH1709	159	−0.7	158	52.1	101	62.6
SY108	162	21.1	183	52.0	99	62.5
YLY6	174	2.4	177	51.7	105	64.8
HLY1511	153	19.8	172	50.5	99	63.8
WSSM	163	24.6	188	43.1	92	57.5
LSD (0.05)	28	34.4	30	5.2	7.9	3.9

In both years, the leaf N concentration was significantly higher than that in the stem and panicle at the heading stage, while the grain N concentration was the highest at maturity only in 2014. At the maturity stage in 2015, the N concentration in the leaf was similar to that in the grain. However, HY549 had the lowest leaf N concentration at maturity in both 2014 and 2015, while YY4949 had the highest N concentration in the leaf and stem at the heading stage in 2015 (Table 5).

**Table 5:** **Nitrogen concentration in various plant organs of the varieties at the heading stage and maturity in 2014 and 2015 at Wuxue County, Hubei Province, China**.

**Variety**	**HD**			**PM**			
	**Nleaf**	**Nstem**	**Npanicle**	**Nleaf**	**Nstem**	**Ngrain**	**Nother**
**2014**							
HY549	2.65	0.85	1.62	0.76	0.52	1.17	0.81
HLY858	2.78	0.88	1.70	1.00	0.49	1.29	0.85
JKY651	2.67	0.84	1.68	1.21	0.49	1.25	0.75
9Y6H	2.63	0.89	1.39	0.91	0.44	1.24	0.77
HLY630	2.75	0.91	1.45	0.96	0.45	1.31	0.89
RFY41	2.55	0.87	1.56	1.09	0.54	1.29	0.84
RY225	2.51	0.80	1.38	0.86	0.51	1.28	0.90
WYH1573	2.65	0.90	1.54	1.19	0.58	1.20	0.63
QY982	2.62	0.77	1.44	0.90	0.40	1.22	0.73
YLY6	2.41	0.80	1.40	0.84	0.41	1.24	0.74
HHZ	2.65	0.86	1.64	1.16	0.52	1.25	0.82
GLY5	2.31	0.69	1.36	0.88	0.44	1.28	0.86
HY73	2.58	0.77	1.42	1.23	0.44	1.26	0.74
ZZ14	2.81	0.95	1.41	1.07	0.56	1.36	0.90
YG29	2.45	0.68	1.36	1.58	0.56	1.45	1.13
LSD (0.05)	0.45	0.24	0.36	0.19	0.13	0.08	0.08
**2015**							
YY4949	3.19	1.12	1.28	1.29	0.58	1.26	0.70
HY549	2.49	0.73	1.25	1.17	0.60	1.15	0.93
CY5727	2.72	0.82	1.26	1.29	0.66	1.26	0.87
JY225	2.94	1.00	1.54	1.41	0.81	1.28	0.87
YY278	2.27	0.70	1.16	1.18	0.54	1.20	0.77
LLYHZ	2.73	0.75	1.27	1.16	0.57	1.22	0.76
HLY630	2.59	0.85	1.31	1.22	0.52	1.21	0.70
MLY143	2.47	0.81	1.28	1.27	0.54	1.24	0.68
LY10	2.62	0.79	1.24	1.35	0.65	1.28	0.80
HHZ	2.93	0.88	1.55	1.46	0.57	1.32	0.89
JY959	2.64	0.79	1.36	1.19	0.55	1.28	0.87
ZH1	2.65	0.78	1.31	1.48	0.56	1.27	0.72
HLY7185	2.62	0.98	1.32	1.37	0.68	1.27	0.75
HH1709	2.93	0.96	1.26	1.23	0.62	1.20	0.85
SY108	2.64	0.83	1.29	1.42	0.65	1.20	0.69
YLY6	2.50	0.76	1.33	1.15	0.53	1.25	0.78
HLY1511	2.61	0.83	1.37	1.24	0.59	1.27	0.71
WSSM	2.77	0.85	1.30	1.28	0.78	1.33	0.81
LSD (0.05)	0.25	0.18	0.11	0.15	0.10	0.11	0.12

### Relationship between NUE and growth traits

**Figure 2:**
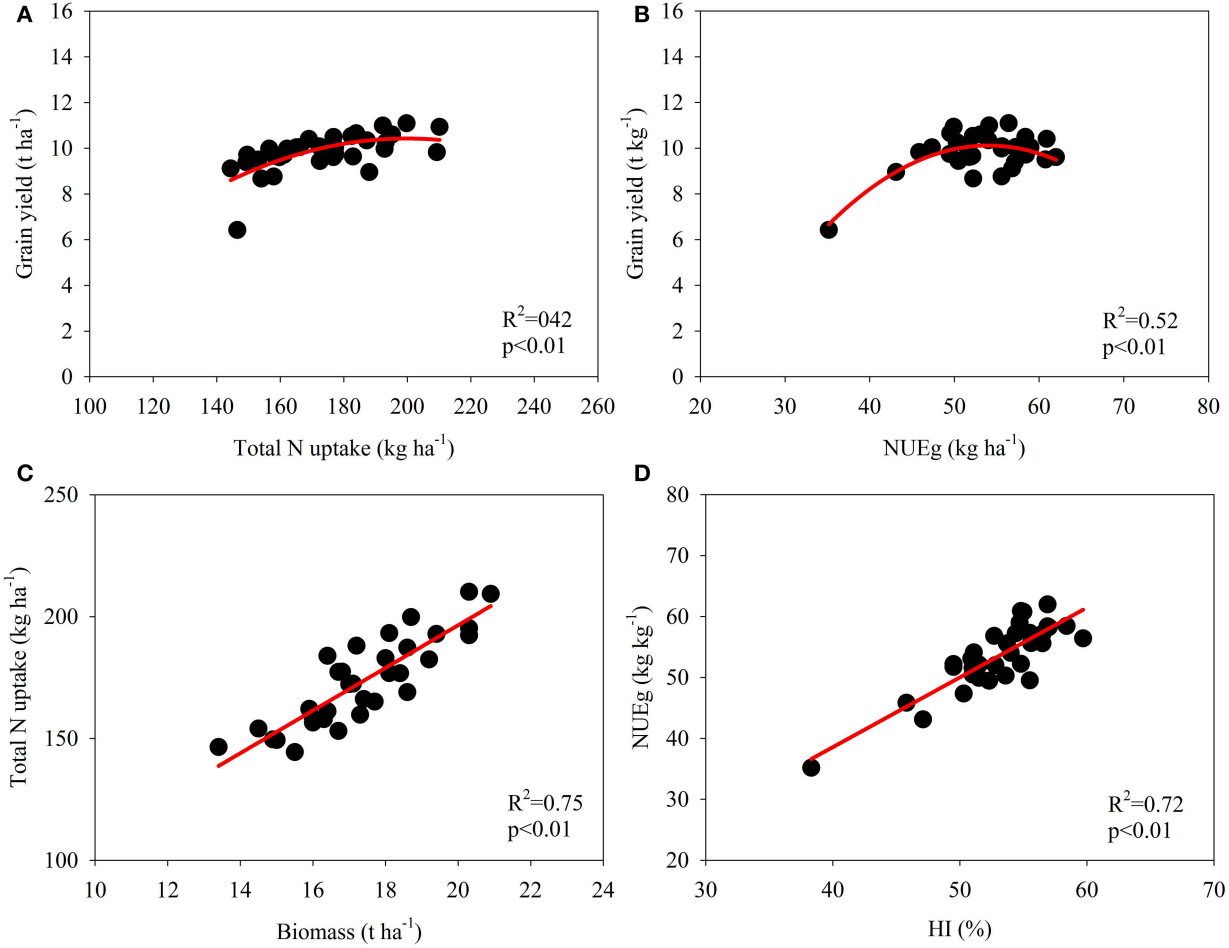
**Correlation between total N uptake at maturity and grain yield (A), NUEg and grain yield (B), biomass at maturity and total N uptake (C), and harvest index (HI) and NUEg (D)**.

**Figure 3:**
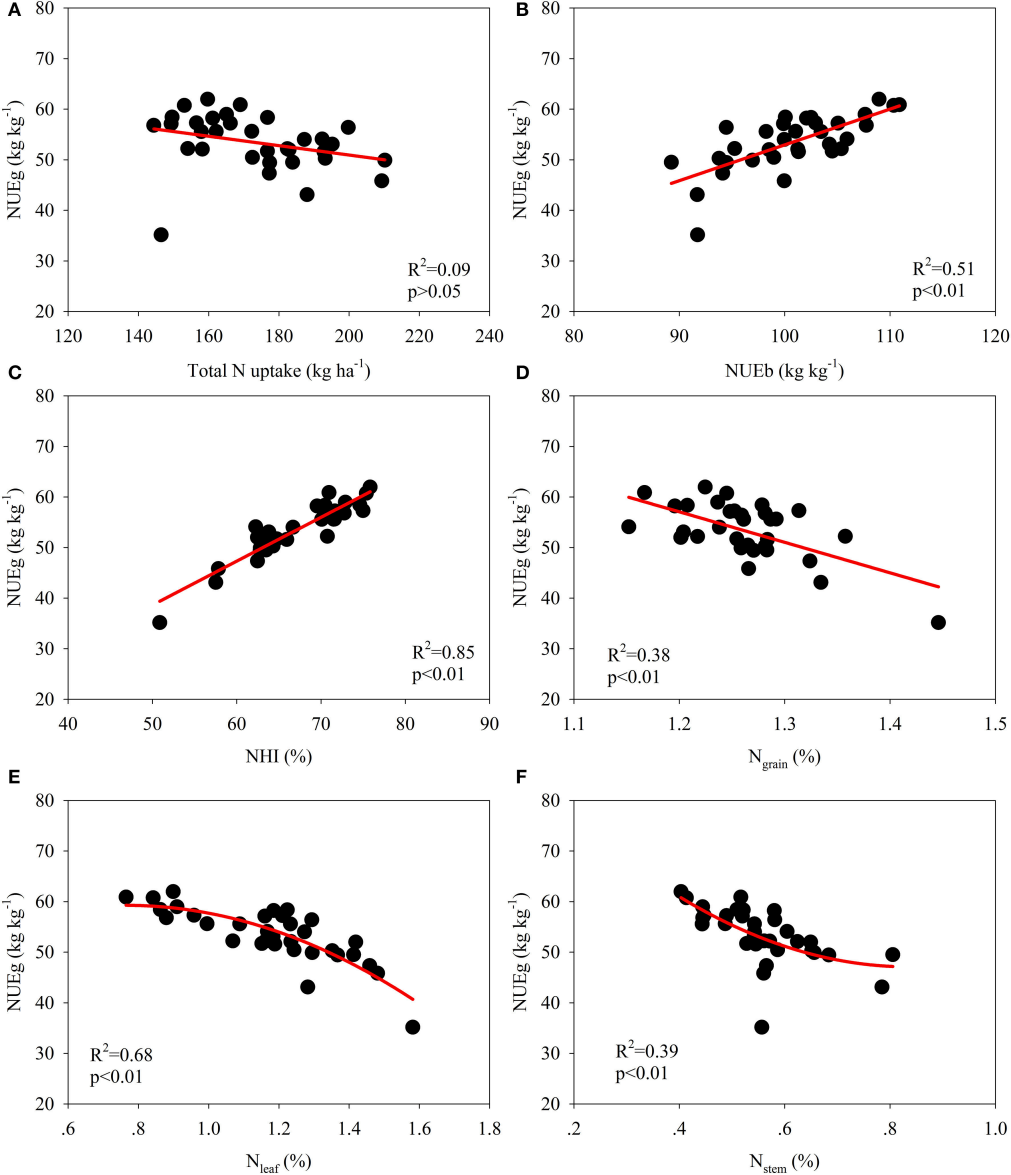
**Correlation among NUE-related parameters and their relationship with the N concentration in various plant organs at maturity**. **(A)** Correlation between total N uptake and NUEg, **(B)** Correlation between NUEb and NUEg, **(C)** Correlation between NHI and NUEg, **(D)** Correlation between N grain and NUEg, **(E)** Correlation between N leaf and NUEg, **(F)** Correlation between N stem and NUEg.

The data for the varieties in 2014 and 2015 were used for correlation analyses to examine the relationship between the NUE-related parameters and growth analyses (Figures 2, 3; Table 6). A significant quadratic relationship was observed between the grain yield and TN_PM_, demonstrating that the grain yield was augmented with an increase in TN_PM_ until 180 kg ha^−1^ (Figure 2A). The significant quadratic relationship between the grain yield and NUEg revealed that improvements in NUEg had no influence on the grain yield when NUEg was higher than 45 kg kg^−1^ (Figure 2B). Improvements in the N uptake capacity and NUEg were accomplished through breeding for high biomass production and HI, respectively (Figures 2C,D). No significant relationship was observed between NUEg and TN_PM_ (Figure 3A). NUEg was significantly and positively correlated with NUEb and NHI, but was negatively correlated with the N concentration in the grain, leaf, and stem (Figures 3B–F). The quadratic relationship between the NUEg and N concentration in leaf and stem suggested that improvements in NUEg were dependent on a decreased leaf N concentration when the NUEg value was lower than 50 kg kg^−1^, while further improvements in NUEg were dependent on a decreased stem N concentration (Figures 3E,F).

**Table 6:** **Correlation between NUE-related parameters and growth-related parameters**.

**Parameters**	**Total N uptake**	**NUEg**	**NUEb**	**NHI**
Total growth duration	0.54^**^	0.17 ns	0.61^**^	−0.38^*^
Daily grain yield	0.36^*^	0.46^**^	0.30 ns	−0.10 ns
Stem No. m^−2^ at heading	0.20 ns	−0.41^*^	0.44^**^	−0.60^**^
Plant height at heading	0.39^*^	0.12 ns	0.26 ns	−0.06 ns
Leaf area index at heading	0.80^**^	−0.27 ns	0.82^**^	−0.76^**^
Specific leaf weight at heading	0.08 ns	−0.30 ns	−0.01 ns	−0.08 ns
Crop growth rate before heading	0.79^**^	−0.18 ns	0.77^**^	−0.67^**^
Biomass at heading	0.70^**^	−0.11 ns	0.72^**^	−0.56^**^
Biomass during grain filling period	−0.79^**^	0.58^**^	−0.89^**^	0.99^**^
Panicle No m^−2^	0.29 ns	−0.37^*^	0.49^**^	−0.62^**^
Spikelets per panicle	−0.07 ns	0.42^*^	−0.34 ns	0.45^*^
Spikelets m^−2^	0.17 ns	−0.14 ns	0.05 ns	−0.06 ns
Grain filling percentage	0.59^**^	0.24 ns	0.72^**^	−0.48^**^
Grain weight	0.01 ns	0.26 ns	−0.01 ns	0.20 ns
Leaf N concentration at heading	0.23 ns	−0.09 ns	0.12 ns	−0.23 ns
Stem N concentration at heading	0.07 ns	0.14 ns	0.01 ns	−0.06 ns
Panicle N concentration at heading	−0.45^**^	0.31 ns	−0.63^**^	0.61^**^

^*^ and ^**^indicate significance at 0.05 and 0.01 probability level.

Many growth traits were significantly and positively correlated with TN_PM_, such as the total growth duration, daily grain yield, plant height at heading, leaf area index at heading, crop growth rate before heading, biomass at heading, and grain filling percentage. However, the biomass during the grain-filling period and the panicle N concentration at heading were negatively correlated with TN_PM_ (Table 6). A significant and positive correlation was observed between the NUEg and biomass during the grain-filling period, spikelets per panicle, and daily grain yield. Most of the growth parameters affected the NUEb or NHI (Table 6).

## Discussion

### Intervarietal difference in NUE

Variations in rice NUE have been studied since the research by Broadbent et al. (1987), who reported significant differences in the NUE of 24 rice genotypes at IRRI. Thereafter, many studies were conducted to examine the rice NUE, showing that TN_PM_ and NUEg ranged from 48 to 130 kg ha^−1^ and 35 to 79 kg kg^−1^, respectively, under irrigated lowland conditions (Tirol-Padre et al., 1996; Singh et al., 1998). Under rainfed lowland conditions, Inthapanya et al. (2000) showed that TN_PM_ ranged from 25.7 to 40.4 kg ha^−1^ and NUEg from 55.1 to 83.8 kg kg^−1^ for 16 genotypes under a N fertilizer rate of 60 kg ha^−1^. Under Mediterranean direct water-seeded conditions, Koutroubas and Ntanos (2003) observed that the NUEg ranged from 60.9 to 90.9 kg kg^−1^ for two *indica* and three *japonica* rice varieties at an N fertilizer rate of 150 kg ha^−1^. In wheat, significant differences in NUE have been examined (Le Gouis et al., 2000). The TN_PM_ and NUEg in wheat ranged from 31 to 264 kg ha^−1^ and 27 to 77 kg kg^−1^, respectively, depending on the N rate, variety, and year (Barraclough et al., 2010; Gaju et al., 2011; Bingham et al., 2012).

In a previous study, we observed that TN_PM_ ranged from 138 to 248 kg ha^−1^, and NUEg ranged from 28.8 to 58.4 kg kg^−1^ for 14 rice mega varieties developed at different ages. Similarly, both TN_PM_ and NUEg were significantly enhanced through the advancements in genetic breeding (Zhu et al., 2016). In the present study, the TN_PM_ of the elite varieties ranged from 144 to 210 kg ha^−1^, which was higher than the values observed for rice at a similar N rate, as previously discussed. NUEg ranged from 35.2 to 60.9 kg kg^−1^, which is consistent with the findings of Tirol-Padre et al. (1996) and Singh et al. (1998), but was lower than the findings of Koutroubas and Ntanos (2003). Koutroubas and Ntanos (2003) reported a grain yield ranging from 6.0 to 8.3 t ha^−1^, thus the relatively high NUEg reflected a lower TN_PM_, which ranged from 76.2 to 124.2 kg ha^−1^. Notably, the grain yield in the present study was significantly higher than the values reported in all of the previous studies, indicating that it is feasible to simultaneously achieve high yield and high efficiency.

### Relationship between grain yield and NUE

Broadbent et al. (1987) evaluated the stability of nine NUE-related parameters using the N^15^ labeling method to rank the genotypes across different seasons, and De Datta and Broadbent (1988) further tested these methods without using isotopically labeled fertilizer to reflect genotypic variations in NUE. These studies showed that the yield and GW/Nt were the most stable parameters reflecting genotypic differences in the NUE of rice. In the present study, the grain yield and daily grain yield were significantly and positively correlated with both TN_PM_ and NUEg (Figure 2, Table 6; Samonte et al., 2006). This finding is consistent with the evidence that genetic improvements in the yield potential improve both TN_PM_ and NUEg (Fischer, 1981; Bingham et al., 2012; Zhu et al., 2016). However, the correlations between the grain yield and TN_PM_ and between the grain yield and NUEg were quadratic (Figures 2A,B; Cassman et al., 1993; Singh et al., 1998). This finding indicated that the increase in grain yield through an increase in TN_PM_ is marginal when TN_PM_ is higher than 150 kg ha^−1^, and this increase is likely to improve NUEg while maintaining a high grain yield.

### Plant traits related with NUE

The N uptake efficiency accounted for 64% of the variation in the NUE at zero N rate, while the NUEg was more significant at a higher N rate (Le Gouis et al., 2000). Gaju et al. (2011) also demonstrated the association between the N uptake efficiency and showed that NUE increased with increasing N limitation. Thus, breeders should select varieties with a high N uptake efficiency for low-yield crops, and varieties with high NUEg for high-yield crops, although it is possible to simultaneously improve the N uptake efficiency and NUEg (Figure 3A; Moll et al., 1982). The following plant traits were associated with TN_PM_ and NUEg.

TN_PM_ could be estimated from primary plant parameters, as this measurement was significantly correlated with the tiller number, spikelet number, main culm panicle node number reflecting the potential tillers and leaves of a plant (Singh et al., 1998; Samonte et al., 2006). Moreover, Singh et al. (1998) observed that varieties with long growth durations had higher TN_PM_ values compared with varieties with medium growth durations. In addition, deeper roots and greater root oxidation activities are important for N uptake at low N rates in both rice and wheat (Foulkes et al., 2009; Worku et al., 2012; Ju et al., 2015). In the present study, TN_PM_ was significantly and positively correlated with total growth duration, plant height at heading, leaf area index at heading, crop growth rate before heading, biomass at heading, and grain filling percentage, but it was negatively correlated with biomass accumulation during the grain filling period and panicle N concentration at heading (Table 6). Consequently, genetically promoting plant growth prior to heading is important for improvements in the TN_PM_ at low N rates.

N utilization efficiency is dependent on the N efficiency of biomass formation, the effect of N on carbohydrate partitioning, nitrate reduction efficiency, and remobilization of N from senescent tissues and storage functions (Foulkes et al., 2009). NUEg was significantly correlated with HI (Figure 2D), as HI was positively and significantly correlated with the dry matter translocation efficiency (Ntanos and Koutroubas, 2002). Mathematically, NUEg is equal to the ratio of the NHI and grain N concentration; thus, the NUEg was positively and significantly correlated with the NHI but was negatively correlated with the grain N concentration (Figures 3C,D). In the present study, the NHI ranged from 57.5 to 75.0%, which is consistent with the values reported in the studies of Tirol-Padre et al. (1996) and Singh et al. (1998). Consequently, it might be possible to further increase the NHI of rice to some extent (Sinclair and Vadez, 2002). Significant negative correlations between the grain N concentration and NUEg have been widely demonstrated among different genotypes in rice and wheat (Singh et al., 1998; Inthapanya et al., 2000; Koutroubas and Ntanos, 2003). Moreover, Cassman et al. (1993) demonstrated that a lower N content grain in rice than that in bread wheat contributes to a higher NUEg in rice, particularly at high yield levels. The straw N concentration explained a large percentage of the genotypic variation in NUEg in the studies of Singh et al. (1998) and Koutroubas and Ntanos (2003). In the present study, we further demonstrated that the variation in NUEg was dependent on the changes in the leaf N concentration at maturity at low NUEg levels, while further increases in NUEg resulted from decreases in the stem N concentration (Figures 3E,F). These results are consistent with the findings in wheat, suggesting that delayed leaf senescence is a key trait for increasing NUEg at low N supply (Foulkes et al., 2009; Gaju et al., 2011). Moreover, NUEg was significantly and positively correlated with biomass accumulation during the grain-filling period, spikelets per panicle and daily grain yield (Table 6).

In conclusion, the present study determined the genotypic variation in NUE among newly developed elite rice varieties in China and demonstrated that genetic improvements in the yield potential under high nutrient input conditions also increased the TN_PM_ and NUEg at a low N supply. The quadratic correlation between the grain yield and TN_PM_ and between the grain yield and NUEg suggests that a further increase in N uptake results in a small increase in grain yield when TN_PM_ is above 160 kg ha^−1^, and it is possible to simultaneously achieve a high grain yield and high NUEg under low N supply. Improvements in the NUE are likely to occur with simultaneous increases in TN_PM_ and NUEg through the improvements in the daily grain yield. Plant traits associated with the rapid crop growth rate prior to heading could be used to increase TN_PM_, while biomass accumulation and a large panicle are essential for improvements in NUEg. Moreover, further improvements in NUEg depend on the increase in the translocation of N from the stems to delay leaf senescence during the grain-filling period.

## Author contributions

LW, SY, LH, and FS conducted the field experiments. SP and FW designed the experiments. SF revised the manuscript. LW analyzed the data, and FW drafted the manuscript.

## Funding

This work was financially supported by grants from the National High Technology Research and Development Program of China (the 863 Project no. 2014AA10A605), the Bill and Melinda Gates Foundation (OPP51587), and the Fundamental Research Funds for the Central Universities (Project 2015BQ002).

### Conflict of interest statement

The authors declare that the research was conducted in the absence of any commercial or financial relationships that could be construed as a potential conflict of interest.
